# Restoring Waning Production of Volatile Organic Compounds in the Endophytic Fungus *Hypoxylon* sp. (BS15)

**DOI:** 10.3390/jof4020069

**Published:** 2018-06-12

**Authors:** Yuemin Wang, James K. Harper

**Affiliations:** Department of Chemistry, University of Central Florida, 4111 Libra Drive, Orlando, FL 32816, USA; Yueminwang@knights.ucf.edu

**Keywords:** endophytic fungi, volatile organic compounds, *Hypoxylon*, production modulators

## Abstract

Certain endophytic fungi belonging to the *Hypoxylon* genus have recently been found to produce volatile organic compounds (VOCs) that have potential relevance as hydrocarbon fuels. Here, a recently discovered *Hypoxylon* sp. (BS15) was demonstrated to also produce VOCs, but with diminished VOC production after an extended period of in vitro growth. Restoring VOC production was partially achieved by growing BS15 in growth media containing finely ground woody tissue from the original host plant (*Taxodium distichum*). In an effort to isolate VOC production modulators, extracts from this woody tissue were made by sequentially extracting with dichloromethane, methanol, and water. Both the dichloromethane and water extracts were found to modulate VOC production, while the methanol extract had no effect. Surprisingly, the woody tissue remaining after exhaustive extraction was also shown to act as a VOC production modulator when included in the growth media, with changes observed in the production of four compounds. This woody tissue also induced production of two compounds not observed in the original BS15 extract. Filter paper had the same modulating effect as exhaustively extracted woody tissue, suggesting the modulation was perhaps due to cellulose degradation products. Overall, this study demonstrated that VOC production in BS15 can be influenced by multiple compounds in the woody tissue rather than a single modulator.

## 1. Introduction

Endophytes are microorganisms, usually fungi and bacteria, that live inside the host plant without showing signs of their presence or causing apparent disease symptoms. The relationship between endophytes and their host plants varies from symbiotic to pathogenic [1]. Typically, tropical areas and rainforests are presumed to have the greatest diversity and abundance of endophytes due to their vast plant diversity [2]. Investigations involving endophytes are of considerable interest, in part due to their production of a remarkable variety of natural products [3,4,5,6]. Although there has been significant focus on endophytes, they remain relatively understudied.

Recently some work has focused on fungi that produce volatile organic compounds (VOCs), including some with potential usefulness as fuels or antimicrobials. For the purposes of this manuscript, VOCs are defined as compounds having sufficient volatility to be separable/mobile on gas chromatography. Table 1 summarizes the presently known fungi that can produce VOCs. Currently, only fungi producing components similar to fossil fuels have been considered to have fuel potential. These compounds include branched alkanes and their derivatives, substituted cyclohexanes, benzenes, alkyl alcohols, aldehydes, and polycyclic aromatic hydrocarbons [7]. For instance, *Gliocladium roseum* produces more than 40 VOCs with fuel potential, such as pentyl, hexyl, heptyl, and octyl alcohols, 3,3,5-trimethyldecane, and other branched hydrocarbons [7]. Among all of the VOCs with fuel potential characterized and reported, 1,8-cineole is a compound of special interest because a 70/30 (*v*/*v*) mixture of petrol/1,8-cineole has performance characteristics similar to petrol with less carbon monoxide emissions [8,9,10]. In 2010, a *Hypoxylon* sp. designated CI-4 was reported as the first non-plant source to produce 1,8-cineole (hereinafter referred to as cineole). More recently, other *Hypoxylon* spp. have also been found to produce cineole [11,12].

An unexpected challenge involving cineole production in CI-4 was the observation that the production gradually decreased over a period of months when the organism was removed from the plant host. This decrease suggested the presence of one of more cineole production modulator compounds in the host plant. Nigg et al. isolated and characterized a modulator in an endophytic *Nodulisporium* species, the imperfect stage of *Hypoxylon* [35]. This modulator was able to restore cineole biosynthesis, and it is likely that similar outcomes can be obtained in other endophytic fungi where production of valuable products decrease over time. In related work, Hassan et al. reported that the treatment of *Hypoxylon* sp. with known epigenetic modulators not only cause phenotypic changes, but also modifies the VOCs production and the bioactivity [34]. All of these prior studies provide new insight into why such a diverse range of VOCs are found in different isolates of *Hypoxylon* spp.

Recently, a *Hypoxylon* sp. not corresponding to any named species was isolated from a bald cypress tree (*Taxodium distichum*) near Orange City, Florida, USA. A complete phylogenetic characterization of this fungus, designated BS15, will be given elsewhere. BS15 produces a variety of VOCs with possible relevance as fuels or antimicrobials. As with other *Hypoxylon* spp., BS15 was found to exhibit a significant decrease in VOC production over time and, motivated by the work of Nigg et al. [35], techniques for restoring VOC production were evaluated. This manuscript describes a process in which extracts from woody tissue of the plant from which BS15 was originally isolated were added to the growth media in an effort to restore VOC production. Serial extractions of the woody tissue were performed with dichloromethane (DCM), methanol, and water. In the following, we describe the changes from each extract and show that the DCM and water extracts, as well as the exhaustively extracted wood tissue, induce production of compounds. These changes were found to be inheritable, and three of the products are shown to differ from those originally produced by BS15, suggesting that the modifications represent epigenetic changes.

## 2. Materials and Methods

Dichloromethane (DCM) and methanol were purchased from Fisher Scientific (Salt Lake City, UT, USA). SiliaPrep C-18 columns were purchased from Silicycle (Quebec City, Canada). Potato dextrose broth (PDB) and agar were purchased from Microtech Scientific (Vista, USA). All reagents were used as received.

The *Hypoxylon* sp. BS15 was isolated from a bald cypress tree (*Taxodium distichum*) near the Saint Johns river near Orange City, Florida, USA. Initially, isolation of the fungus followed the procedures of Tomsheck et al. [11]. This involved treating branches with 70% ethanol, further sterilizing the wood in a flame, and then drying in a sterile laminar-flow hood. Outer tissue was cut away using a sterile knife blade and a square section of inner tissue was then placed on water agar. Any fungal hyphae growing out from the sample were transferred onto different plates of potato dextrose agar (PDA). One such sampling resulted in isolation of BS15.

Growth of BS15 samples without added modulators (i.e., extracts from *T. distichum*) was accomplished in PDB prepared by adding 2.4 g of potato dextrose broth to 100 mL purified water in a 500 mL Erlenmeyer flask. The flask was sealed with aluminum foil and autoclaved for 15 min to sterilize. A culture of BS15 growing on PDA was then added to the sterile broth, and it was resealed with aluminum foil and left to grow for 30 days in the lab at room temperature without stirring. The resulting broth was then vacuum filtered twice with Whatman Grade 4 filter paper to remove all particulates.

Extractions of woody tissue involved taking approximately 210 g of shredded cypress wood and extracting with 400 mL DCM for 90 min with stirring. This process was repeated two times. The DCM was then removed on a rotatory evaporator (Buchi Rotavapor R-205, Buchi, New Castle, DE, USA) at reduced pressure. The cypress wood was further extracted with methanol, and finally with water, using 400 mL of each solvent. In order to simulate the extractable components in a wood matrix, all three fractions were dripped onto filter papers, and the filter paper was allowed to completely dry. This process was repeated several times until the filter paper contained the desired mass of the extracted components. These filter papers were cut into pieces and added to 100 mL of the PDB medium in a 500 mL Erlenmeyer flask, and this flask was inoculated with BS15. As a control, filter paper containing no extractables was added to the PDB/BS15 media. As a final test, the exhaustively extracted cypress wood was also added to PDB. All flasks were then sealed with aluminum foil and autoclaved for 15 min. After cooling, BS15 was added to the sterile broth, and the resulting solution was allowed to grow for 30 days at room temperature without stirring.

Media used to evaluate the influence of the DCM, methanol, and water extracts on BS15 were prepared by removing a sample of the fungi grown in a solution containing PDB plus *T. distichum* extracts after 30 days of growth and transferring it onto a petri dish. Serial weekly transfers onto PDA were then performed over a period of 4 weeks to ensure that all changes in VOC production ultimately observed were epigenetic changes, and that exogenous contaminants from the extractable components were rigorously removed.

Isolation of VOCs involved solid phase extraction of the growth media on a C-18 stationary phase (500 mg). First, a C-18 cartridge (particle size: 40–63 µm) was washed with 5 mL of methanol, and then with 5 mL of water (three times). A total of 100 mL of filtered fungal broth was then passed through the column under vacuum. The column was washed with 15 mL of water to remove polar components (e.g., salts), and the column was dried by drawing air through the column for 30 min. The column was then eluted by passing 1.5 mL of methanol through the column to yield a clear brown solution. The eluent was filtered using a 0.22 µm syringe filter prior to gas chromatography/mass spectrometry (GC/MS) analysis. This solid-phase extraction methodology differs from the solid-phase microextraction (SPME) methodology usually employed when evaluating fungal VOCs. The methodology was employed in order to more efficiently retain compounds having low vapor pressure, and which may be missed by SPME.

The GC/MS method used was similar to that of Strobel et al. [36]. The instrument used was a Finnigan TraceGC Ultra with Trace DSQ detector (Thermo Scientific) and a Restek Rtx-225 capillary column (cyanopropyl-methyl/phenyl-methyl polysiloxane, 50/50, 30 m × 0.25 mm, film thickness 0.25 µm) (Restek, Bellefonte, PA, USA). The carrier gas was ultra-high purity helium with a 1.5 cm^3^/min constant flow rate and an initial column head pressure of 77 kPa. The injector was set to 250 °C with a 1 µL injection volume using splitless injection mode. The column oven temperature was initially 45 °C and held for one minute, followed by a 10 °C/min ramp to 100 °C, where the temperature was held for 5 min. Finally, the temperature was increased by 5 °C/min to 200 °C and held for 7 min. The detector was set at 280 °C and set to scan 50–650 *m*/*z*. Data acquisition and processing were performed on Xcalibur software. Identification of compounds was made via library comparison using National Institute of Standards and Technology (NIST) database. In all the GC/MS analyses describe herein, quantities of individual compounds detected are not reported because many of the compounds are unknowns. This ambiguity prevents the construction of calibration curves required for quantitation.

## 3. Results and Discussion

**Figure 1:**
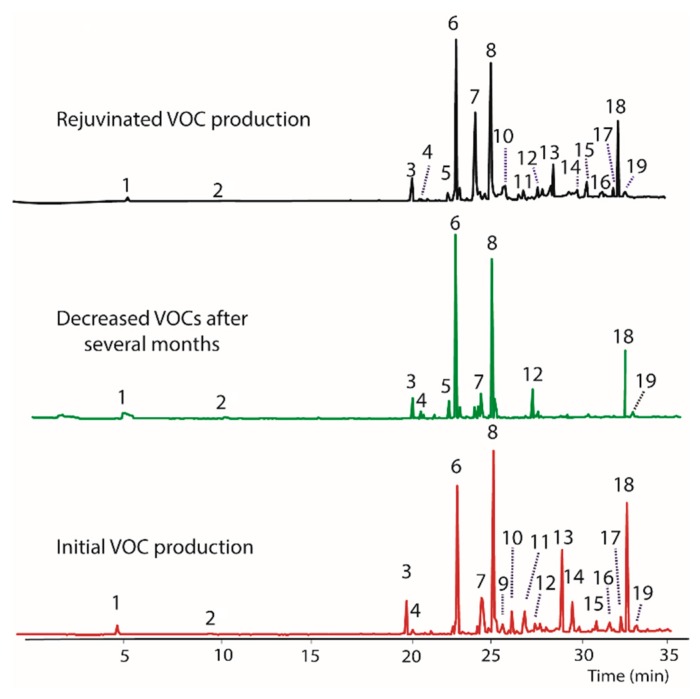
A gas chromatogram illustrating VOCs produced by BS15 showing the original production of VOCs immediately after isolation of BS15 (**bottom**) and decreased production after growing in the lab for several months (**middle**). Nominal mass of each peak and tentative identities are listed in Table 2. Production of VOCs was restored to varying extents (**top**) by growing BS15 on PDA containing finely ground woody tissue from the *Taxodium distichum* the fungus was originally isolated from.

The endophytic fungus BS15 was selected for study based on the observation that some of the compounds produced had a distinctive odor, which indicated production of volatile compounds. A GC/MS analysis of the original BS15 revealed a number of VOCs (Table 2). Unfortunately, the production of several compounds decreased with time in the absence of the host plant (Figure 1). The nominal masses of all compounds were obtained and five compounds were tentatively identified. A more complete characterization of the compounds present will be given elsewhere. When BS15 with diminished VOC production was transferred back to PDA containing woody tissue from the host plant (i.e., finely ground *Taxodium distichum* tissue), production of most VOCs was restored, albeit to varying degrees (Figure 1, top plot). The ability to restore VOC production in BS15 suggested the presence of a modulator compound or multiple modulators in the host plant.

## 4. Decreased VOC Production in BS15 after Extended In Vitro Growth

In order to investigate modulators from the host plant that restores production of VOCs in BS15, serial organic solvent extraction of woody tissue was made using DCM followed by methanol and then water. Each extract was then tested for its ability to restore VOC production in BS15. Since some of the extracted compounds were insoluble in the growth media, the extracted solutions were dripped onto filter paper and then air dried. Filter paper was employed to simulate the woody matrix of the original tissue. This process was repeated until the desired mass of extract had been loaded onto the filter paper (see Section 2). Growth media (PDB) was then prepared, and the filter paper impregnated with extractable compounds was included in the media. The filter paper was cut into strips of approximately 1” × ¼” to give a uniform distribution in solution. In each case, a control was also prepared containing filter paper with no extract added. The exhaustively extracted wood was also evaluated by including it in the growth media. The impact of each extract on production of volatiles is discussed below.

## 5. Assessing the Influence of DCM Extract/Filter Paper on VOC Production

**Figure 2:**
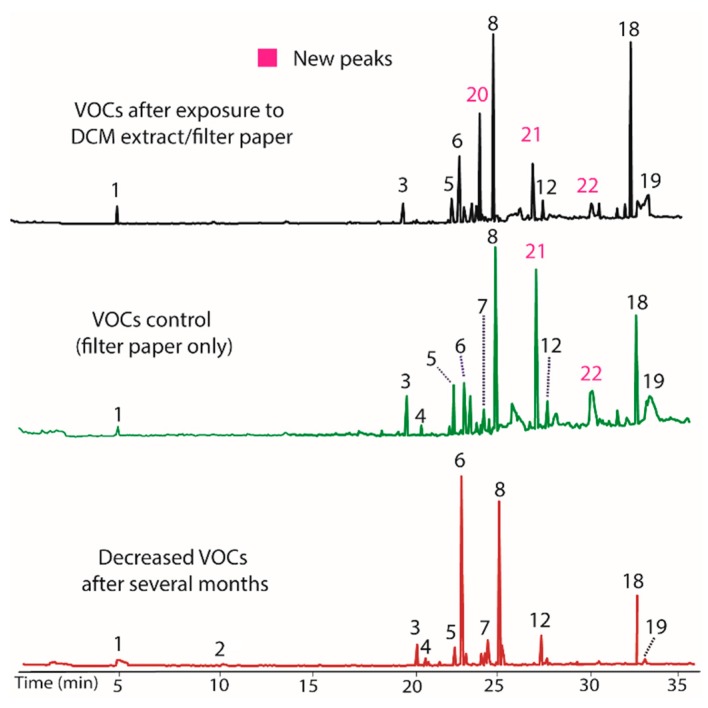
Gas chromatograms showing decreased VOCs production by BS15 after growing in vitro for several months (**bottom**). Adding BS15 to growth media (PDB) containing DCM extract/filter paper induced production of new compounds **20**, **21**, and **22** (**top**), and altered the production of compounds **6** and **18**. The **middle** plot demonstrates that filter paper containing no DCM extract also induces production of compounds **21** and **22** when included in growth media Nominal mass of each peak and their tentative identities are listed on Table 3.

A culture of BS15 grown in a PBD medium containing DCM extract/filter paper was found to alter the VOCs produced by inducing the production of three new compounds. Specifically, the peaks labeled **20**, **21**, and **22** in Figure 2, with respective nominal masses of 112, 216, and 154, were observed only after addition of the DCM extract, and thus appear to represent an epigenetic change to BS15. Surprisingly, the control containing only filter paper also induced production of compounds **21** and **22**. In both cases, these changes in VOC production were inheritable, and persist over several generations. Indeed, Figure 2 represents BS15 VOCs obtained from tissue removed from the DCM/filter paper media, then plated onto PDA, followed by weekly transfers onto PDA for one month, and finally regrown in PDB. In other words, Figure 2 represents a BS15 culture that was three generations removed from the initial DCM/filter paper treatment. Careful inspection of the chromatograms showed that the DCM extracts also increased production of peak **18** and decreased production of **6**. Overall, it appeared that DCM contained a modulator that altered production of peaks **6** and **18**, and created the ability to produce **20**. Remarkably, the filter paper appeared to be solely responsible for the production of compounds **21** and **22**, as discussed below. Isolation of individual modulator compounds from the DCM extract was not performed due to insufficient mass of DCM extract.

## 6. Evaluating the Influence of Methanol and Water Extracts on VOC Production in BS15

**Figure 3:**
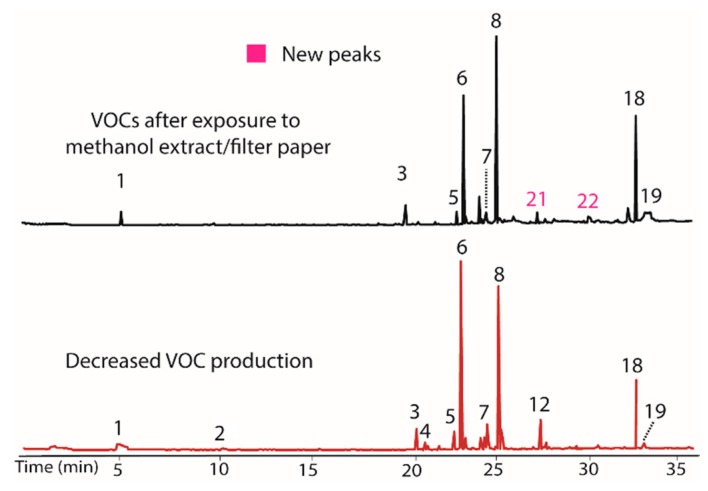
Chromatograms showing the negligible influence of the methanol extract/filter paper on VOC production in BS15. The new peaks (**21** and **22**) observed upon treatment (**top**) also occur in the control containing only filter paper. Their occurrence is therefore attributed to a change from the filter paper rather than the presence of VOC production modifiers extracted by methanol. The **bottom** plot illustrates BS15 after prolonged in vitro growth.

The influence of both the methanol and water extracts from *T. distichum* on BS15 were also evaluated using the process described above for the DCM extract. The methanol extract/filter paper produced almost no change, with the exception that peaks **21** and **22** were again observed. Chromatograms illustrating VOC production before and after addition of methanol extract/filter paper are shown in Figure 3. In this case, the control containing only filter paper also induced production of **21** and **22**. The production of these compounds also occurred in the DCM extract and their occurrence is thus attributed to the filter paper as a VOC production modifier rather than any compounds extracted by methanol.

**Figure 4:**
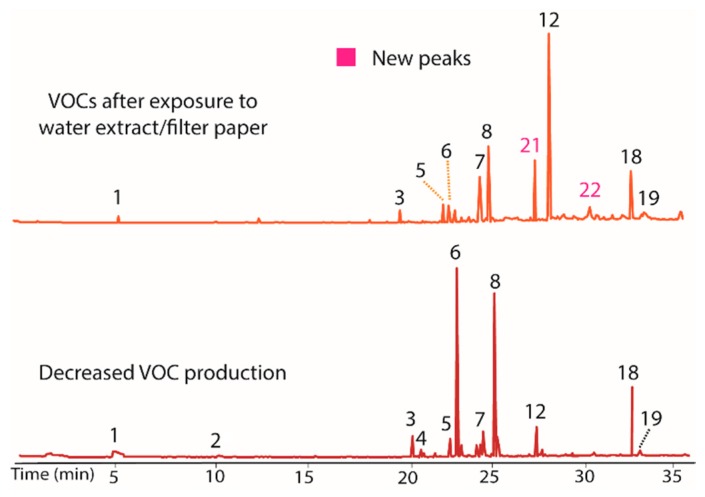
Chromatograms showing the influence of the water extract/filter paper of VOC production of BS15. Peaks **7**, **12**, and **18** increased upon exposure to the water extract while **6** decreased (**top**) versus BS15 with diminished VOC production (**bottom**). The water extract thus appeared to contain a modulator of VOC production. Peaks **21** and **22** were again observed to occur in both the water extract and in the control containing only filter paper.

The water extract/filter paper was also evaluated (Figure 4) using the process described above. This extract increased production of compounds **7**, **12**, and **18**, while decreasing the quantity of **6**. The water extract thus likely contained a VOC production modulator. As in the other extracts, peaks **21** and **22** were again observed in both the extract/filter paper and in the control, strengthening the conclusion that filter paper induces their production. All changes from the water extract were inheritable.

## 7. The Influence of Exhaustively Extracted *T. distichum* Wood on VOC Production in BS15

**Figure 5:**
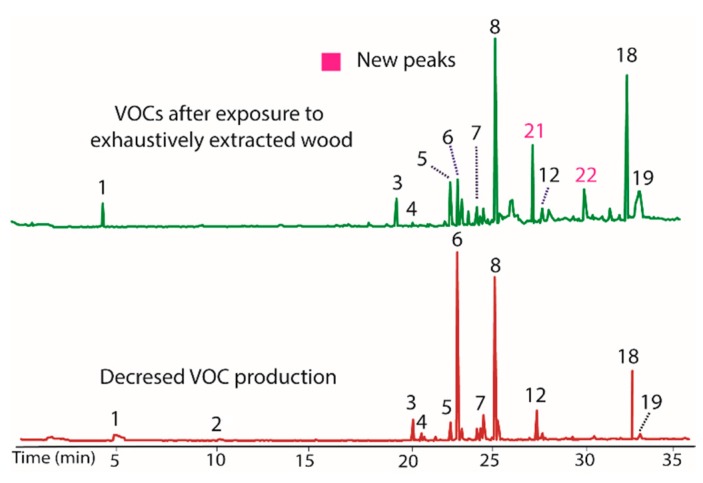
Chromatograms showing the influence on VOC production (**top**) of the addition of exhaustively extracted woody tissue of *T. distichum* to growth media (PDB). Peaks **5** and **18** increased, while **7** and **12** decreased versus BS15 after prolonged in vitro growth (**bottom**). Peaks **21** and **22** appeared as prominent components in the top plot, supporting the contention that these peaks were induced by cellulose containing materials.

**Figure 6:**
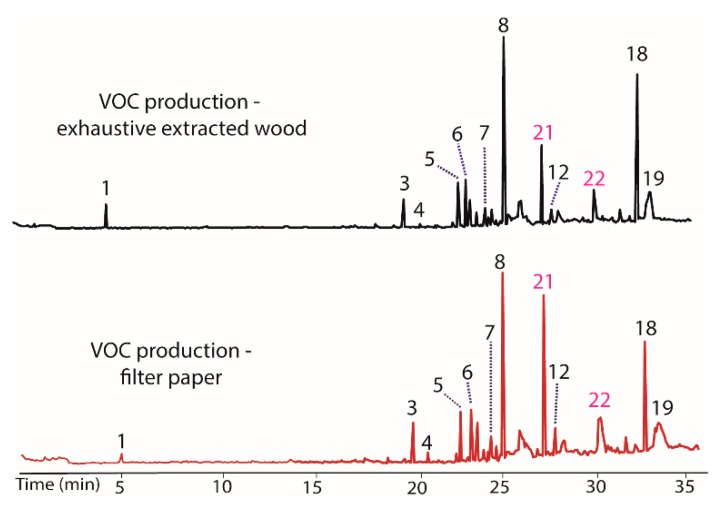
Chromatograms illustrating the changes in the production of VOCs by BS15 following exposure to filter paper (**bottom**) and to exhaustively extracted woody tissue from branches of *T. distichum* (**top**).

As a final test of potential modulators of VOC production in BS15, the finely ground extracted woody tissue of *T. distichum* was evaluated. This unusual step was taken because the filter paper was repeatedly found to induce production of peaks **20** and **21** in prior extracts, and it was of interest to see if other cellulose contain materials could have the same effect. The extracted wood was observed to decrease production of peaks **7** and **12**, while it increased **5** and **18** (Figure 5). Of greatest interest was the observation that peaks **21** and **22** appeared as prominent peaks, supporting the contention that these peaks were induced by cellulose containing materials. A comparison of the woody tissue to the filter paper controls from each extract is illustrated in Figure 6. The remarkable similarity in the changes induced by filter paper and those induced by exhaustively extracted wood from *T. distichum* indicated that a common production modulator is involved.

A direct comparison of each extract and its influence on VOC production is summarized in Table 4, and shows that the influence of DCM, water, and extracted wood (or equivalently, filter paper) clearly differed. This result indicated that multiple modulators were involved in VOC production as opposed to a single modulator.

An important question regarding the influence of modulators on VOC production is how long the fungus with compromised VOC production should be exposed to media containing ground plant tissue or extractable compounds in order to restore VOC production. In the case of BS15, we observed that continuous exposure to the woody tissue or extracts/filter paper for three generations was sufficient to restore production. For the purposes of this study, a transfer of the fungus was made each week to new media containing modulators (i.e., extracts). Thus, three generations correspond to three weeks. In all cases, the fungus was removed from the media containing extractables or wood and grown for a week to maturity before transfer to PDB to ensure that all exogenous contaminations were removed. Similarly, it was important to evaluate how long the fungus with restored production could grow in vitro before VOC production began to decrease. In BS15, a significant decrease in VOC production was observed after eight generations (8 weeks).

## 8. Conclusions

The research demonstrated that waning production of volatile organic compounds in an endophytic *Hypoxylon* sp. (BS15) can be partially restored by re-exposure to chemical constituents contained in the DCM and water extracts of the woody tissue of *T. distichum*. Surprisingly, the exhaustively extracted woody tissue also induced changes in VOC production from BS15 by causing production of two compounds never observed in the original growth media, and in altering production of four other compounds. Filter paper alone can also produce this change, suggesting that the differences are epigenetic changes, and that cellulose or its degradation products were the active component in altered VOC production rather than other compounds (e.g., lignans). The cellulose-based modification of production may have been caused by hydrolysis of the woody tissue or filter paper, which could create water soluble carbohydrates, and further study of this effect is needed. It is notable that carbohydrates are known to alter gene expression in some bacteria [37], thus there is a precedent for this change in BS15 from cellulose containing materials. An interesting parallel to this observation of activity in cellulose, a material that is essentially insoluble, is a prior study [35] reporting that breakdown products from lignin (i.e., ferulic acid and vanillin) also act as VOC production modulators in a closely related endophytic fungus.

It is notable that the large solubility difference between the water and DCM extracts suggests that the production modulator in DCM differed from that found in water. Thus, it is likely that more than one modulator effectively influenced VOC production in BS15. At present, insufficient amounts of the DCM and water extracts were available to allow isolation of individual compounds, and future work will focus on identifying compounds in these extracts involved in VOC modulation.

Solid-phase extraction was used in this study, rather than the more commonly employed approach of solid-phase microextraction, in order to more effectively include compounds having lower vapor pressures. One possible limitation to this approach is that compounds having high vapor pressures may be underrepresented. Further study is underway to directly compare these two techniques.

## Figures and Tables

**Table 1:** A list of fungi presently known to produce volatile organic compounds.

Species	Site Isolation	Extraction Method *^a^*	No. of VOCs Detected	Reference
*Aspergillus fumigatus*	New Jersey, USA	SPME	>10	[13]
*A. niger*	New Jersey, USA	SPME		[13]
*A. tubingensis*	New Jersey, USA	SPME		[13]
*A. niger*	Malaysia	LLE	>295	[14]
*Fusarium armeniacum*	New Jersey, USA	SPME	>10	[13]
*F. graminearum*	New Jersey, USA	SPME		[13]
*F. oxysporum*	New Jersey, USA	SPME		[13]
*F. proliferatum*	New Jersey, USA	SPME		[13]
*F. culmorum*	Belgium	SPME	>10	[15]
*F. langsethiae*	Russia	SPME	>40	[16]
*F. sibiricum*	Russia	SPME		[16]
*F. poae*	Russia	SPME		[16]
*F. sporotrichioides*	Russia	SPME		[16]
*Metarhizium anisopliae*	New Jersey, USA	SPME	>5	[13]
*Mucor racemsus*	New Jersey, USA	SPME	>10	[13]
*Penicillium chrysogenum*	New Jersey, USA	SPME	>10	[13]
*P. citreonigrum*	New Jersey, USA	SPME		[13]
*P. commune*	New Jersey, USA	SPME		[13]
*P. corylophilum*	New Jersey, USA	SPME	>10	[13]
*P. crustosum*	New Jersey, USA	SPME		[13]
*P. glabrum*	New Jersey, USA	SPME		[13]
*P. pinophilum*	New Jersey, USA	SPME		[13]
*P. polonicum*	New Jersey, USA	SPME		[13]
*P. sclerotiorum*	New Jersey, USA	SPME		[13]
*P. steckii*	New Jersey, USA	SPME		[13]
*P. sumatrense*	New Jersey, USA	SPME		[13]
*Nodulisporium*	Canary Islands, Ecuador, Thailand, Nicaragua, South Australia, Colombia, and Wetlands of Florida	SPME	>40	[17]
*Muscodor albus*	Honduras, Thailand, and Ecuador	SPME	>20	[18]
*M. crispans*	Bolivian Amazon basin	SPME	>15	[19]
*M. kashayum*	India	SPME	>20	[20]
*M. strobelii*	India	SPME	>14	[21]
*M. darjeelingensis*	India	SPME	>20	[22]
*M. tigerii*	India	SPME	>20	[23]
*M. suthepensis*	Thailand	SPME	>25	[24]
*M. musae*	Thailand	SPME	>15	[24]
*M. oryzae*	Thailand	SPME	>15	[24]
*M. equiseti*	Thailand	SPME	>15	[24]
*M. sutura*	Colombia	SPME	>20	[25]
*M. fengyangensis*	China	SPME	>20	[26]
*Myrothecium inunduatum*	India	SPME	>30	[27]
*Bionectria ochroleuca*	India	LLE	>5	[28]
*Ampelomyces*	Japan	SPME	>5	[29]
*Phoma*	Japan	SPME	>5	[29]
*Cladosporium*	Japan	SPME	<5	[29]
*Phomopsis*	Ecuador	SPME	>10	[30]
*Gliocladium roseum*	Northern Patagonia	SPME	>40	[7]
*Beauveria bassiana*	Montana, USA	SPME	6	[31]
*Ascocoryne sarcoides*	Northern Patagonia, UK, Germany, France, Norway, and Canada	SPME	>100	[32]
*A. cylichnium*	Norway, Switzerland	SPME		[32]
*A. solitaria*	Netherlands	SPME		[32]
*Schizophylum commune*	Chile	SPME	10	[33]
*Hypoxylon*	Thailand, Spain	SPME	>15	[34]

*^a^* The abbreviations LLE and SPME denote liquid-liquid extraction and solid phase micro-extraction, respectively.

**Table 2:** A GC/MS analysis of the VOCs produced by BS15.

Peak	R.T. (min)	Tentative Identify	Mol. Mass
**1**	4.75	unknown	70
**2** *^a^*	9.41	1,8-cineole	154
**3**	19.15	unknown	142
**4**	19.78	unknown	120
**5**	21.84	unknown	126
**6** *^a^*	22.35	Phenyl ethyl alcohol	122
**7** *^a^*	23.85	2,3-naphthalenediamine	158
**8**	24.51	unknown	182
**9**	24.75	Unknown	184
**10**	25.87	unknown	220
**11** *^a^*	26.58	Phenylacetic acid	136
**12**	27.62	Unknown	298
**13** *^a^*	28.72	Diethyl phthalate	222
**14**	29.52	unknown	297
**15**	30.72	unknown	213
**16**	31.47	unknown	334
**17**	32.15	unknown	213
**18**	32.63	unknown	192
**19**	33.27	unknown	314

*^a^* Assignment confidence for peaks **2**, **6**, **7**, **11**, and **13** are, respectively, 86%, 89.3%, 82.7%, 76.4%, and 94%. No other peaks correspond to compounds in the NIST database.

**Table 3:** Compounds detected by GC/MS from BS15 modified by treatment with DCM extract/filter paper.

Peak	R.T. (min)	Tentative Identify	Mol. Mass
**1**	4.75	Unknown	70
**2**	9.41	1,8-Cineole	154
**3**	19.15	Unknown	142
**4**	19.78	Unknown	120
**5**	21.84	Unknown	126
**6**	22.35	Phenyl ethyl alcohol	122
**7**	23.85	2,3-Naphthalenediamine	158
**8**	24.51	Unknown	182
**9**	24.75	Unknown	184
**10**	25.87	Unknown	220
**11**	26.58	Phenylacetic acid	136
**12**	27.62	Unknown	298
**13**	28.72	Diethyl phthalate	222
**14**	29.52	Unknown	297
**15**	30.72	Unknown	213
**16**	31.47	Unknown	334
**17**	32.15	Unknown	213
**18**	32.63	Unknown	192
**19**	33.27	Unknown	314
**20** *^a^*	23.76	3-methyl-2,5-furandione	112
**21**	26.85	Unknown	216
**22** *^a^*	29.82	4,4′-thiobis-benzeneamine	154

*^a^* Assignment confidence for peaks **20** and **22** are, respectively, 67% and 71%.

**Table 4:** A summary of compounds influenced by different extracts of *T. disticum ^a^*.

Extract	Increased Production	Decreased Production	New Compounds Produced
DCM	**18**	**6**	**20, 21, 22**
Methanol	**None**	**None**	**21, 22**
Water	**7, 12, 18**	**6**	**21, 22**
Wood, extracted	**5, 18**	**7, 12**	**21, 22**
Filter paper	**5, 18**	**7, 12**	**21, 22**

*^a^* All analyses of VOC production were made on fungi from the fourth generation after initial exposure to the extract or woody tissue/filter paper.
